# Warm Springs

**Published:** 1875-09

**Authors:** W. F. Westmoreland

**Affiliations:** Warm Springs, N. C.


Warm Springs, N. C., Aug. 11, 1875.

Dear Journal:

Here I am, upon the banks of the beautiful French Broad, away from the din, dust and bustle of the city, surrounded by all that is lovely in the way of scenery, with invigorating mountain air, devoid of all the doubtful odors; and I am better—yes, I may say I am myself again.

Just one week ago I arrived here, jaded and worn—yes, sick from the toils, responsibilities, anxieties and perplexities incident to the life of an active practitioner, and to-day I feel like a new being. And what has wrought this great change in so short a time? Is it the result of the warm baths, which are so agreeable and soothing to to the nervous system, or the sulphur, saline, or chalybeate waters which we find here? or is it this bracing mountain air, mountain mutton, mountain trout, etc., with complete rest, both mental and physical? I attribute my rapid restoration to the latter, as I had no specific malady, but was simply exhausted from over-work during the extreme hot weather of July.

But while I attribute my restoration to rest, cool, pure mountain air, beautiful scenery, etc., I would not be understood as belonging to that class of physicians who deny the efficacy of mineral waters in the cure of disease. Had I felt otherwise, I have seen enough in the short time I have been here to convince me that in certain classes of affections much may be accomplished by the judicious use of the baths, and in others by drinking the mineral waters.

The suspicion and incredulity with which the mass of the profession look upon the reported results of mineral waters is attributable in a large degree to the wholesale and "cure-all" style of advertising so frequently pursued by the proprietors of watering-places, and which smacks of quackery to the extent that it can but be repulsive to the scientific physician. But, however repulsive the surroundings, should it deter us from doing what is so plainly our duty in the premises? Should we not investigate more thoroughly this great question, that we may be able to say with some degree of certainty what may be expected from this, to the patient, more popular and pleasant mode of treating disease? It is too often the case that we send our patients to the "springs" with the hope that the surroundings at least will be beneficial, without any reference whatever to the character of the waters, which in many instances are in no way adapted to their diseases. This is all wrong, and frequently results in great injury to our patients.

In my next I propose to discuss the character of the waters as well as the surroundings of this place, and make some suggestions as to their action and the class of cases likely to be benefited by a sojourn here.

Truly yours, W. F. Westmoreland.

